# Sex-specific hippocampal microstructural alterations in 11–12-year-old adolescents with a history of mild traumatic brain injury

**DOI:** 10.3389/fnbeh.2026.1766772

**Published:** 2026-05-08

**Authors:** Jiyoung Ma, Deborah A. Yurgelun-Todd, Erin C. McGlade

**Affiliations:** 1 Department of Psychiatry, Huntsman Mental Health Institute, University of Utah School of Medicine, Salt Lake City, UT, United States; 2 George E. Wahlen Department of Veterans Affairs Medical Center, VISN 19 Mental Illness Research, Education and Clinical Center, Salt Lake City, UT, United States

**Keywords:** diffusion MRI, early adolescence, hippocampus, mild traumatic brain injury (mTBI), restriction spectrum imaging, sex differences

## Abstract

**Introduction:**

Mild traumatic brain injury (mTBI) is common in children and adolescents and frequently accompanied by transient cognitive and emotional disturbances. While pediatric mTBI can shape subsequent brain maturation, its impact on neurodevelopmental trajectories remains poorly understood, particularly regarding hippocampal microstructure, a region critical for memory and highly vulnerable to brain injury.

**Methods:**

Using restriction spectrum imaging (RSI) data from the Adolescent Brain Cognitive Development Study (ABCD Study^®^; *n* = 4,399), this study aimed to characterize hippocampal microstructural alterations in adolescents (aged 11–12 years) with a history of mTBI and to examine their associations with verbal learning and memory performance on the Rey Auditory Verbal Learning Test (RAVLT). Sex-stratified analyses were performed considering potential sex differences in vulnerability and responses to brain injury. History of mTBI was assessed using the modified Ohio State University TBI Screen-Short Version, a retrospective parent report.

**Results:**

Male adolescents with a history of mTBI exhibited higher hippocampal restricted normalized diffusion (RND) than their peers without mTBI (Bonferroni-corrected *p* = 0.02), whereas no group differences were observed in females. Within the male mTBI group, higher hippocampal RND was positively associated with both immediate (*p* = 0.01) and delayed (*p* = 0.04) recall scores on the RAVLT, suggesting potential adaptive or reparative neuroplastic processes following injury.

**Discussion:**

These findings demonstrate sex-specific hippocampal microstructural alterations in young adolescents with a history of mTBI, highlighting potential neuroplastic adaptations during development. Although causal inference is limited given the cross-sectional design of the analysis, and mTBI history was based on retrospective parent report, the results underscore the importance of considering sex differences in recovery mechanisms and may inform future efforts to identify neuroimaging biomarkers that predict recovery trajectories and guide targeted interventions.

## 1 Introduction

Mild traumatic brain injury (mTBI), often referred to as concussion, is caused by external mechanical forces such as falls, blasts, or rapid acceleration-deceleration and can result in brief loss of consciousness (LOC ≤ 30 min), memory loss (≤ 24 h), or altered mental status (Lumba-Brown et al., 2018; Ruff et al., 2009). It affects approximately 7% of children under 17 years of age (Black and Zablotsky, 2021), with an estimated 1–2 million cases occurring annually in the United States (Bryan et al., 2016). mTBI has been associated with a range of acute cognitive and emotional symptoms, including decreased attention, memory difficulties, frustration, and depressive mood (Barlow et al., 2010; Eisenberg et al., 2014), and it is also associated with challenges in school, which can have lifelong effects beyond cognitive deficits (Waltzman et al., 2024). Most children who experience mTBI show clinical recovery within several weeks (Barlow et al., 2010; Eisenberg et al., 2014), likely reflecting neuroplastic and compensatory processes in the developing brain. However, the potential consequences of pediatric mTBI on brain maturation and neurodevelopmental trajectories remain insufficiently characterized.

When external forces are applied to the head, the brain can collide with the skull, which may result in axonal stretching and shearing and subsequent neurometabolic cascades characterized by ionic disturbances and neuroinflammatory processes, ultimately disrupting normal brain function (Giza and Hovda, 2014; Johnson et al., 2013). One of the most common clinical symptoms following mTBI is decreased memory functions. Interestingly, the hippocampus, a key structure involved in memory processing, has been suggested to be particularly vulnerable to mTBI due to its anatomical location within the middle cranial fossa and its proximity to the sphenoid bone, clinoid processes, and tentorium cerebelli (Bigler, 2023). Moreover, the hippocampus is highly sensitive to secondary pathophysiological cascades following injury, including excitotoxicity, mitochondrial dysfunction, and inflammation (Bigler, 2023). Several animal studies of mTBI have demonstrated biomolecular and neurophysiological changes in the hippocampus, including cellular degeneration (Girgis et al., 2016; Ustaoglu et al., 2021) and neuroinflammation (Wiegand et al., 2022), and have linked these alterations to post-injury cognitive and memory deficits (Girgis et al., 2016). Previous neuroimaging studies of mTBI have reported hippocampal microstructural disruptions in the chronic phase (Leh et al., 2017), as well as structural alterations in adjacent regions, including the entorhinal cortex (Khoury et al., 2024) and parahippocampal gyrus (Sussman et al., 2017).

Most studies examining hippocampal microstructural alterations following mTBI have been conducted with adults, whereas corresponding evidence in pediatric populations remains limited. Adolescence is a critical period of neurodevelopment, during which responses to the effects of mTBI occur alongside ongoing brain maturation. Understanding how mTBI-related brain alterations in adolescents diverge from normative developmental trajectories therefore requires dedicated investigation. Given that this period represents a sensitive window that shapes long-term brain development, early and targeted interventions are especially important. Identifying non-invasive neuroimaging markers associated with mTBI and post-injury symptoms in adolescents could inform the development of more effective strategies to reduce clinical and neurodevelopmental consequences.

Alterations in subcortical structures such as the hippocampus have traditionally been examined using conventional structural magnetic resonance imaging (MRI) metrics (e.g., volume, morphometry) and diffusion MRI indices (e.g., mean diffusivity, fractional anisotropy). However, recent advances in neuroimaging now permit the estimation of more subtle microstructural characteristics that are not captured by conventional structural or diffusion measures. In particular, restriction spectrum imaging (RSI) applies a multicompartment mathematical model to multi-shell diffusion data across a broader range of b-values, whereby the diffusion signal is decomposed into restricted (i.e., intracellular) and hindered (i.e., extracellular) components (White et al., 2013). Quantification of the restricted component provides metrics that reflect brain cytoarchitecture, including cellularity and neurite-related microstructural features. Specifically, increases in RSI-derived restricted diffusion are thought to correspond to greater neurite density, dendritic complexity, and enlarged cell body within the typical diffusion length scale in human imaging (~10 μm) (White et al., 2013). Further separating restricted diffusion into isotropic (RNI) and directional (RND) components provides additional insight into tissue organization. RNI increases with larger neuronal or glial cell bodies and less coherent neurite organization, including tangled neurites, whereas RND increases with highly aligned, unidirectional fiber architecture, such as tightly packed pyramidal neurons with coherent projections or white matter bundles. The hippocampus is densely cellular and also contains organized white matter pathways, including the fimbria and alveus. Accordingly, RNI and RND may each provide insight into hippocampal microstructural organization. Notably, RSI-derived restricted diffusion metrics may be particularly sensitive to detecting such microstructural alterations after brain injury (Hutchinson et al., 2018) and have also demonstrated sensitivity to subtle structural characteristics of the developing brain (Genc et al., 2017; Palmer et al., 2022).

This study examined microstructural characteristics, measured with RSI, in the hippocampus and its associated white matter tracts in adolescents with and without a history of mTBI. We focused on the hippocampus considering their key role in memory, vulnerability to injury, and capacity for neuroplasticity, making this circuitry a promising target for therapeutic and mechanistic research. We analyzed RSI data from the Adolescent Brain Cognitive Development Study^SM^ (ABCD Study®), an ongoing prospective cohort of over 11,000 children from 21 data collection sites across the United States (Volkow et al., 2018). The study initiated baseline assessments of 9–10-year-olds between 2016 and 2018 and has since conducted biennial neuroimaging, thereby enabling large-scale analysis with minimized age-related confounds. The data release used for this analysis (release 5.1) provided neuroimaging from participants aged 11–12 years, along with detailed lifetime mTBI history and memory-related cognitive assessments. This dataset offers an unparalleled opportunity to investigate microstructural alterations associated with lifetime mTBI history and their links to neuropsychological outcomes during early adolescence, prior to major developmental transitions.

We hypothesized that (1) adolescents with a history of mTBI would show hippocampal microstructural reorganization, reflected by increased restricted diffusion, consistent with histological evidence of post-injury alterations, including cytoskeletal and dendritic remodeling as well as glial and inflammatory processes that may increase tissue cellularity and neurite density (Girgis et al., 2016; Ustaoglu et al., 2021; Wiegand et al., 2022); (2) these changes would be associated with memory performance; and (3) these associations might differ between males and females, given prior evidence of sex differences in susceptibility to brain injury and in subsequent recovery trajectories (Dolle et al., 2018; Gupte et al., 2019; Newell et al., 2020; Song et al., 2024). Experimental work suggests that such differences may reflect underlying biological differences between males and females, including variation in neuroinflammatory responses and cellular vulnerability following injury or stress (Dolle et al., 2018; Islam et al., 2024; Newell et al., 2020). Sex differences in neurodevelopmental trajectories may also shape patterns of brain vulnerability and recovery across development (Kaczkurkin et al., 2019). Accordingly, pooling males and females may obscure meaningful sex-specific patterns. We therefore conducted a sex-stratified analysis to characterize potential sex-specific patterns of hippocampal microstructural alterations associated with mTBI.

Recent ABCD studies applying advanced diffusion models, including RSI, have begun to identify sex-specific microstructural alterations following pediatric mTBI (Nishat et al., 2023, 2024; Guberman et al., 2024), suggesting that mTBI may differentially influence neurodevelopment in males and females. In contrast to the current study, these investigations have largely focused on major white matter pathways, and hippocampal microstructure remains relatively understudied, representing an important gap in understanding sex-specific neurodevelopmental vulnerability following pediatric mTBI.

## 2 Materials and methods

### 2.1 Participants

This study used data from the ABCD Study data release 5.1 (https://doi.org/10.15154/z563-zd24). The ABCD Study protocol was approved by the Institutional Review Board (IRB) at each data collection site, with central IRB approval at the University of California, San Diego. The ABCD Study sample was largely recruited through local school systems. Participants were excluded if they had schizophrenia, moderate to severe autism spectrum disorder, intellectual disability, alcohol/substance use disorder, contraindications to magnetic resonance imaging, or a history of severe TBI. Additional exclusion criteria included preterm birth (gestational age < 28 weeks or birth weight < 1,200 g) and lack of fluency in English. The parent/caregiver provided written informed consent, and all youths provided verbal assent. Full details of the enrollment procedures for the ABCD Study are available elsewhere (Garavan et al., 2018).

**Figure 1:**
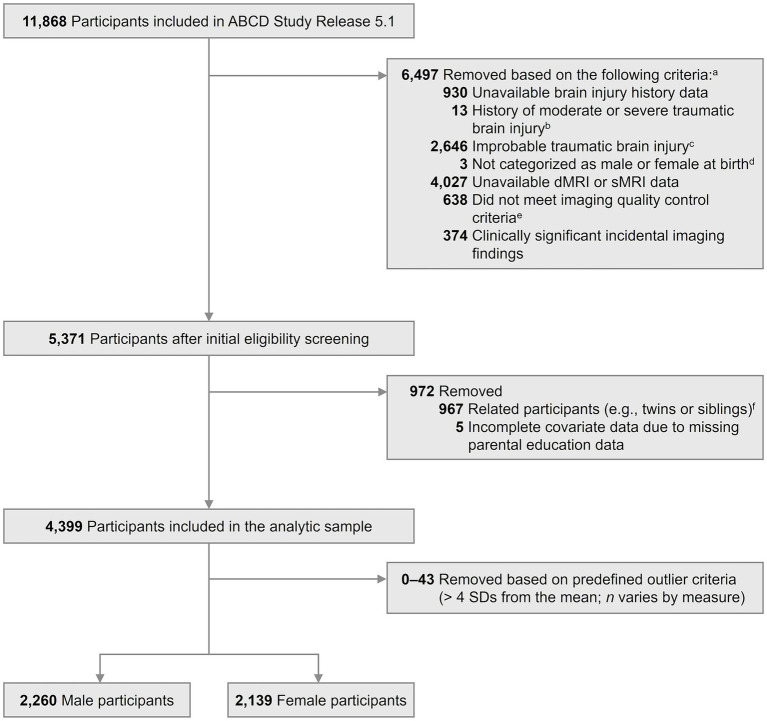
Flow diagram of participant inclusion. ^a^ Participants could meet more than one exclusion criterion. ^b^ Moderate-to-severe traumatic brain injury was considered a head or neck injury involving loss of consciousness lasting more than 30 min, as assessed by the Modified Ohio State University TBI Screen-Short Version (Corrigan and Bogner, 2007). ^c^ Participants classified as having an improbable mTBI (i.e., a history of head or neck injury without loss of consciousness or memory loss/feeling dazed, as assessed by the Modified Ohio State University TBI Screen-Short Version; Corrigan and Bogner, 2007) were excluded, as they did not meet criteria for either the mTBI or control group. ^d^ Participants not categorized as male or female at birth were excluded due to the small sample size. ^e^ Imaging data flagged as not recommended for analysis based on ABCD quality control procedures were excluded. ^f^ The ABCD cohort includes both singletons and twins/siblings as part of its sample design (~9,780 single births and ~1720 twins; Garavan et al., 2018), indicating inherent familial clustering in the dataset. Given the small family cluster sizes and potential convergence issues when adding an additional family-level random effect, we retained one participant per family to ensure independence and model stability. ABCD Study, Adolescent Brain Cognitive Development Study; dMRI, diffusion magnetic resonance imaging; sMRI, structural magnetic resonance imaging; SD, standard deviation.

In the current study, we aimed to examine differences in restricted diffusion measures between adolescents with and without a lifetime history of mTBI occurring before age 12. We therefore excluded adolescents with clinically significant incidental findings or missing quality-controlled structural or diffusion imaging data from the ABCD Study two-year follow-up assessment. Observations with missing covariate data (*n* = 5) were excluded using listwise deletion. Furthermore, we randomly retained only one youth per family in the analysis to account for potential non-independence within families and to improve model stability. Extreme outliers in continuous variables (i.e., > 4 standard deviations from the mean) were also excluded to avoid potential measurement errors (*n* = 0 to 23 depending on the measure). The final sample included a total of 4,399 adolescents (Figure 1). Sample characteristics are summarized in Table 1.

**Table 1:** Demographics and clinical characteristics of participants.

Variables	All (*n* = 4,399)	Lifetime history of mTBI	
		No (*n* = 4,045)	Yes (*n* = 354)
Sex			
Male, *n* (%)	2,260 (51.4)	2,039 (50.4)	221 (62.4)
Female, *n* (%)	2,139 (48.6)	2,006 (49.6)	133 (37.6)
Age, years	11.9 ± 0.6	11.9 ± 0.6	12.0 ± 0.6
Parental education, *n* (%)			
≤ High school	601 (13.7)	574 (14.2)	27 (7.6)
Some college	1,079 (24.5)	996 (24.6)	83 (23.5)
Bachelor’s degree	1,112 (25.3)	1,005 (24.8)	107 (30.2)
Postgraduate degree	1,607 (36.5)	1,470 (36.3)	137 (38.7)
Race/ethnicity, *n* (%)			
Asian	100 (2.3)	96 (2.4)	4 (1.1)
Black	610 (13.9)	584 (14.4)	26 (7.3)
Hispanic	902 (20.5)	849 (21.0)	53 (15.0)
White	2,336 (53.1)	2,106 (52.1)	230 (65.0)
Other^a^	451 (10.3)	410 (10.1)	41 (11.6)
Rey auditory verbal learning test^b^			
Verbal learning	44.4 ± 9.0	44.3 ± 9.1	45.2 ± 8.3
New learning	5.1 ± 1.4	5.1 ± 1.4	5.2 ± 1.4
Immediate recall	9.8 ± 2.7	9.7 ± 2.7	9.9 ± 2.8
Delayed recall	9.1 ± 2.9	9.1 ± 2.9	9.3 ± 2.9
Consolidated information	0.81 ± 0.20	0.81 ± 0.20	0.80 ± 0.19
Crystallized intelligence^c^	103.4 ± 17.1	103.3 ± 17.2	105.1 ± 15.7

Data are presented as mean ± standard deviation unless otherwise specified. ^a^ Other includes participants who are American Indian, Alaskan Native, Native Hawaiian or other Pacific Islander, or of multiple races. ^b^ Data were missing for 156 (3.5%) to 259 (5.9%) participants, depending on the variable. ^c^ Age-corrected standardized scores from the National Institutes of Health (NIH) Cognition Toolbox are presented. Data were missing for 882 (20.1%) participants. mTBI, mild traumatic brain injury.

### 2.2 Mild traumatic brain injury (mTBI)

Adolescents with a lifetime history of mTBI were identified using the Modified Ohio State University TBI Screen-Short Version (Corrigan and Bogner, 2007), a parent-report measure of youths’ lifetime history of head or neck injury. This questionnaire includes items capturing the occurrence of head or neck injuries, including potential injury mechanisms (e.g., falls, motor vehicle accidents, blast exposures), age at the time of injury, and whether the event involved LOC or memory loss. Adolescents with a lifetime history of mTBI (referred to as the mTBI group) were defined as those classified as possible mTBI (TBI without LOC but with memory loss or feeling dazed) or mTBI (TBI with LOC lasting less than 30 min) before age 12. Consistent with prior mTBI research, both possible and definite mTBI categories (i.e., with or without LOC) were included to capture the broader spectrum of mild injuries (Lumba-Brown et al., 2018; Ruff et al., 2009). Adolescents who did not experience a head or neck injury were assigned to the No TBI group.

### 2.3 Magnetic resonance imaging (MRI) data acquisition

Magnetic resonance imaging data were collected using harmonized protocols across multiple sites and scanner platforms (3 T Siemens, General Electric, and Philips) with 32-channel head coils (Casey et al., 2018). T1-weighted anatomical images were acquired at 1-mm isotropic resolution using a three-dimensional (3D) magnetization-prepared rapid acquisition gradient echo (MPRAGE) sequence. Diffusion-weighted images (DWIs) were obtained using a multi-shell, multiband echo-planar imaging (EPI) sequence with the following parameters: 1.7-mm isotropic resolution, acceleration factor = 3, seven *b* = 0 s/mm^2^ frames and 96 gradient directions with six directions at *b* = 500 s/mm^2^, 15 directions at *b* = 1,000 s/mm^2^, 15 directions at *b* = 2000 s/mm^2^, and 60 directions at *b* = 3,000 s/mm^2^. Preprocessing of all DWIs was conducted by the ABCD Data Analysis and Informatics Core. This included eddy current correction, motion correction, and B0 and gradient nonlinearity distortion correction (Hagler et al., 2019). Mean (SD) framewise displacement, rotation, and translation were 1.2 (0.4) mm, 0.36 (0.21) rad, and 0.84 (0.25) mm, respectively. These motion parameters did not differ between the mTBI and No TBI groups (all *p* > 0.4).

### 2.4 Restriction spectrum imaging (RSI) measures

The RSI model was applied to diffusion data to characterize voxel-level diffusion profiles. The RSI separates water diffusion into two compartments, including intracellular (restricted) and extracellular (hindered) compartments, based on the intrinsic diffusion properties of water molecules in the brain. It also provides an estimate of the fraction of anisotropic restricted diffusion of water in a voxel, using mixtures of spherical harmonic basis functions. For this study, we used the RSI-derived metrics of isotropic (RNI) and directional (RND) restricted diffusion. RNI was computed from the norm of the zeroth-order spherical harmonic coefficient and represents isotropic restricted diffusion in a given voxel (White et al., 2013). RND was derived from the norm of the second- and fourth-order spherical harmonic coefficients of the restricted fraction and reflects anisotropic restricted diffusion (White et al., 2013). These metrics range from 0 to 1, with higher values indicating greater isotropic (RNI) and anisotropic (RND) signal fractions. All RSI values were obtained from the tabulated data provided by the ABCD study and were standardized to Z-scores using the sample mean and standard deviation to enable comparison across brain regions.

#### 2.4.1 Hippocampal and subcortical regions

For the hippocampus, RNI and RND values were extracted as the primary measures of interest. The same measures were also extracted from other subcortical structures (e.g., amygdala, thalamus, caudate, putamen, pallidum, nucleus accumbens) for comparison. The subcortical structures were labeled with the Aseg atlas (Fischl et al., 2002).

#### 2.4.2 Memory-associated white matter pathways

To provide complementary information on broader circuit-level microstructure, mean RND values were also examined in five white matter tracts associated with hippocampal-medial temporal and fronto-temporal networks (fornix, parahippocampal cingulum, uncinate fasciculus, inferior longitudinal fasciculus, and arcuate fasciculus), given their established roles in verbal learning and memory (Huang et al., 2021; Shekari and Nozari, 2023). The corpus callosum was additionally included for comparison. White matter tracts were segmented by AtlasTrack (Hagler et al., 2009).

### 2.5 Subcortical volumetric measures

To assess macroscopic structural alterations beyond microstructural measures, subcortical volumes were additionally examined. Volumes based on the Aseg atlas were estimated using FreeSurfer v5.3 (Fischl et al., 2002; Hagler et al., 2019). Regional volumes were similarly standardized to *Z*-scores to facilitate comparison across regions.

### 2.6 Memory performance

Verbal learning and memory were assessed at the imaging visit using the Rey Auditory Verbal Learning Test (RAVLT; Strauss et al., 2006), administered under standardized ABCD Study procedures (Luciana et al., 2018). In this task, participants were presented with a list of 15 words (List A) across five learning trials and asked to recall the words after each presentation. A second list of 15 different words (List B) was then presented to assess interference, followed by immediate recall of words from List A. After a 30-min interval during which other tasks were administered, participants were again asked to recall words from List A to assess longer-term retention. In the ABCD Study, the RAVLT is administered using a computerized and automated protocol to minimize potential site-level differences in task administration (Luciana et al., 2018). In addition, alternate forms of the RAVLT are used across study waves (e.g., different word lists at baseline and follow-up visits) to reduce practice effects across repeated assessments. The primary performance measures used in this study included the total correct recollections across the five learning trials (verbal learning), immediate recall after interference (immediate recall), average of the first trial and List B interference trial (new learning), delayed recall after the 30-min interval (delayed recall), and the proportion of information retained over time, calculated as delayed recall relative to the total correct responses on the last learning trial (consolidated information). Higher scores indicate better verbal learning and memory performance (Strauss et al., 2006).

### 2.7 Statistical analysis

#### 2.7.1 Primary analyses

All statistical analyses were performed in Stata SE version 18.0. Mixed-effects regression models were used to examine group differences in neuroimaging measures (RSI metrics and subcortical volumes) and clinical outcomes by sex. Planned sex-stratified analyses were conducted to characterize sex-specific patterns of group differences. Group × sex interaction terms were included to formally test sex differences in group effects. All models included age, parental education, and race/ethnicity as covariates. Total intracranial volume was included to account for potential global brain size effects on regional diffusion measures (Eikenes et al., 2023; Takao et al., 2011). Parental education and race/ethnicity were obtained from parent-reported demographic questionnaires. As all imaging measures were standardized using the sample mean and standard deviation, the resulting regression coefficients (*B*) are interpretable as effects expressed in standard deviation units. Group differences in RSI and volumetric measures were Bonferroni corrected for the number of variables tested. To address potential clustering of data (e.g., multiple scanners, study sites), scanner serial number nested within study site was included as a random effect in the mixed-effect models.

In additional models, pubertal development status (PDS; Petersen et al., 1988) was included as a covariate to evaluate whether the main sex-specific findings were robust to developmental stage.

#### 2.7.2 Subgroup sensitivity analyses

To assess robustness and account for potential heterogeneity in injury characteristics, we conducted sensitivity analyses stratified by LOC status (No LOC vs. LOC < 30 min), age at injury (early [0–7 years] vs. later [8–12 years] childhood), and emergency department visit history. Consistent with our *a priori* hypotheses, these analyses examined hippocampal restricted diffusion and memory performance within each subgroup. Detailed results are reported in Supplementary materials.

#### 2.7.3 Correlations with memory performance

Associations between hippocampal restricted diffusion measures and memory performance measures were examined using mixed-effects regression models with the same covariates and random effects as in the primary analyses. Interaction terms were included to examine whether mTBI status moderated the relationship between hippocampal microstructure and memory performance, relative to the expected association observed in adolescents in the No TBI group. As a sensitivity analysis, models were additionally adjusted for age-corrected crystallized intelligence from the NIH Toolbox Cognitive Battery (NIHTB-CB; Akshoomoff et al., 2013) to account for individual differences in premorbid cognitive ability (Holdnack et al., 2017). Because RAVLT outcome measures reflect related aspects of verbal memory and are not statistically independent, correction for multiple comparisons across these outcomes was not applied (Sullivan and Feinn, 2021).

## 3 Results

In this sample, 8.0% of participants (*n* = 354) reported a history of mTBI before age 12, with a higher prevalence in males (9.8%) than in females (6.2%; χ^2^ = 18.8, *p* < 0.001). Among those with a history of mTBI, 55.9% were hospitalized or seen in an emergency room following the injury. Falls were the most common cause (58.8%), followed by motor vehicle accidents (8.8%). The mean age at injury was 7.7 ± 3.2 years, and a mean time since injury was 4.3 ± 3.2 years. No sex differences were found in these TBI-related characteristics (all *p* > 0.30; Table 2). Regarding cognitive outcomes, no significant group differences were observed in memory performance assessed by RAVLT or NIHTB-CB crystallized intelligence in either sex (all *p* > 0.07).

**Table 2:** Head injury characteristics.

Variables	All (*n* = 354)	Male (*n* = 221)	Female (*n* = 133)	*p* ^a^
Age at first injury, years	7.7 ± 3.2	7.8 ± 3.0	7.5 ± 3.4	0.36
Time since first injury, years	4.3 ± 3.2	4.2 ± 3.0	4.5 ± 3.4	0.32
Symptoms^b^, *n* (%)				
Loss of consciousness	90 (25.4)	54 (24.4)	36 (27.1)	0.58
Memory loss/feeling dazed	306 (86.4)	193 (87.3)	113 (85.0)	0.53
Hospitalized or treated in an emergency room following an injury, *n* (%)	198 (55.9)	119 (53.8)	79 (59.4)	0.31
Injury from a fall, *n* (%)	208 (58.8)	130 (58.8)	78 (58.6)	0.97
Injury from a motor vehicle accident, *n* (%)	31 (8.8)	22 (10.0)	9 (6.8)	0.30

Data are presented as mean ± standard deviation unless otherwise specified. ^a^ Sex differences in head injury characteristics were analyzed using *t*-tests for continuous variables and chi-square tests for categorical variables. ^b^ Some adolescents reported more than one symptom.

### 3.1 mTBI-related differences in RSI-derived measures and subcortical volumes

**Figure 2:**
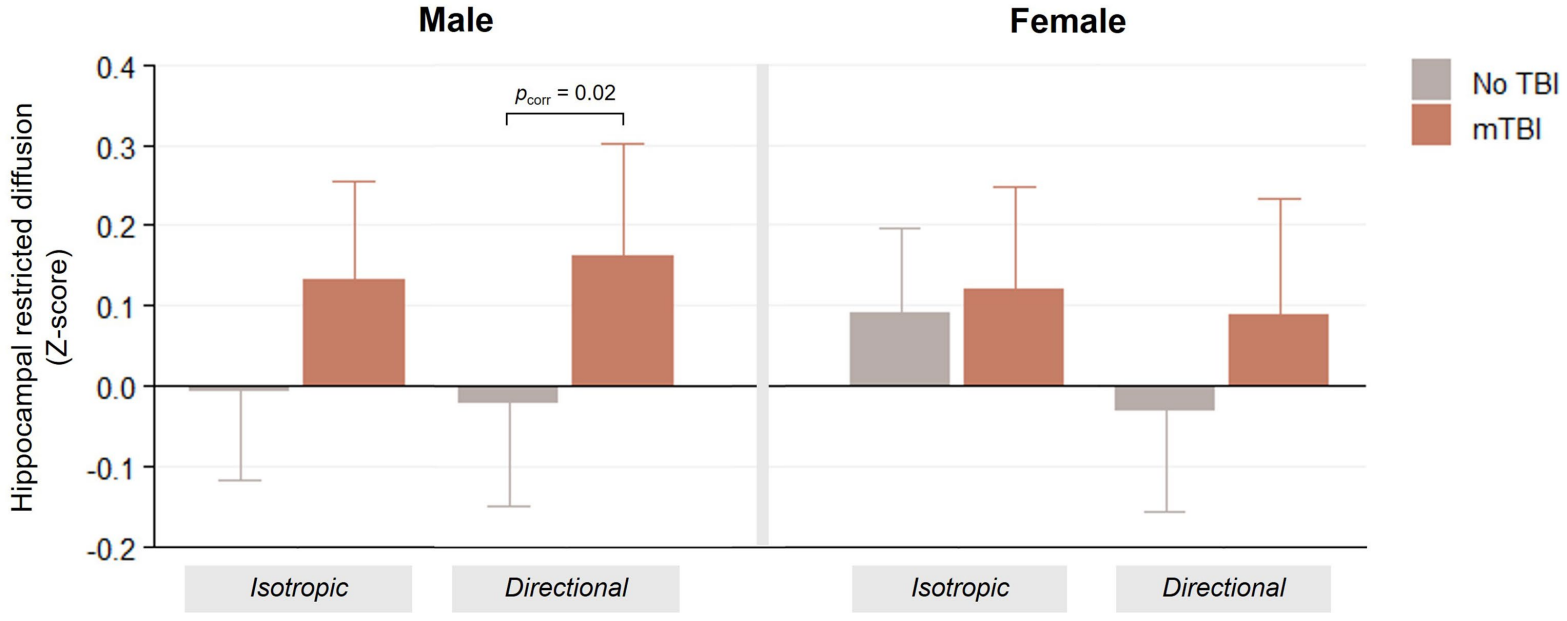
Differences in hippocampal restricted diffusion measures between female and male adolescents with and without mTBI. Group differences were tested within each sex using a mixed-effects regression model, including age, parental education, race/ethnicity, and total intracranial volume as covariates and scanner and study site as random effects. The bar heights represent the predicted mean, and the error bars represent its standard error. Bonferroni-corrected *p-*value (*p_corr_*) is shown. mTBI, mild traumatic brain injury.

Male adolescents with mTBI exhibited greater hippocampal RND compared to those without TBI (*B* [95% confidence interval] = 0.18 [0.06–0.30], *z* = 3.0, *p*_uncorr_ = 0.003; *p*_corr_ = 0.02) (Figure 2). A similar pattern was observed for hippocampal RNI; however, this effect did not survive Bonferroni correction (*B* [95% confidence interval] = 0.14 [0.02–0.26], *z* = 2.3, *p*_uncorr_ = 0.02; *p*_corr_ = 0.15). These differences were not observed in female adolescents (all *p*_uncorr_ > 0.1; *p*_corr_ > 0.8). No significant group differences were observed in other subcortical structures for either RND or RNI (all *p*_uncorr_ > 0.1; *p*_corr_ > 0.9; see Supplementary Table 1 for complete statistics).

No significant group differences were observed in hippocampal-associated white matter tracts (fornix, parahippocampal cingulum, uncinate fasciculus, inferior longitudinal fasciculus, and arcuate fasciculus) or in the comparison tract (corpus callosum), with generally small effect sizes (all *p*_uncorr_ > 0.2; *p*_corr_ = 1.0; Supplementary Table 2). Volumes of the hippocampus and other subcortical structures likewise did not differ between groups in either sex (all *p*_uncorr_ > 0.1; *p*_corr_ = 1.0; Supplementary Table 3).

Group × sex interaction terms were not significant for any brain regions (all *p* > 0.07). Findings were unchanged after additional adjustment for pubertal development status.

### 3.2 Subgroup sensitivity results

Sensitivity analyses stratified by LOC status, age at injury, and emergency department visit history yielded broadly similar directional patterns, although statistical significance was attenuated in some subgroups, likely reflecting reduced power. Age-at-injury analyses suggested that earlier mTBI exposure in females was associated with lower RAVLT performance, whereas no comparable pattern was observed in males. Full subgroup results are provided in Supplementary Tables 4–6.

### 3.3 Associations between hippocampal RSI metrics and memory performance

**Figure 3:**
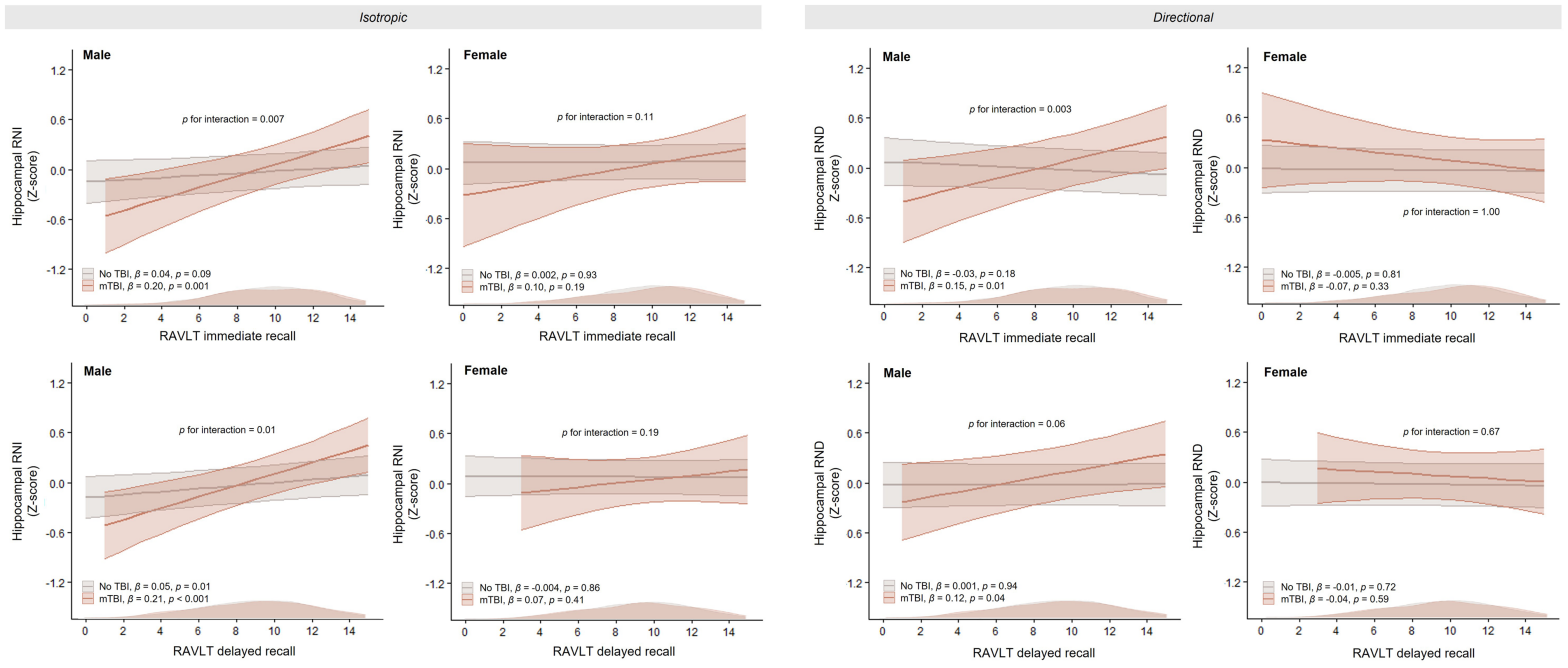
Association between hippocampal restricted diffusion measures and RAVLT immediate and delayed recall scores by mTBI group and sex. Associations were tested using a mixed-effects regression model with age, parental education, race/ethnicity, and total intracranial volume as covariates, and scanner and study site as random effects. Solid lines represent the regression lines, and shaded areas represent 95% confidence intervals. Distributions of RAVLT scores by group are shown along the axis. RNI, restricted normalized isotropic diffusion; RND, restricted normalized directional diffusion; RAVLT, Rey Auditory Verbal Learning Test; mTBI, mild traumatic brain injury.

We further examined the relationship between hippocampal restricted diffusion and memory performance measures. In male adolescents with mTBI, both hippocampal RNI and RND were significantly associated with RAVLT immediate and delayed recall, accompanied by significant group × RAVLT interaction effects (Figure 3). In females, neither interaction effects nor within-group associations between hippocampal restricted diffusion and RAVLT outcomes reached significance (all *p* > 0.10).

To determine whether these associations were independent of premorbid IQ (Holdnack et al., 2017), we conducted sensitivity analyses controlling for crystallized IQ. Adjustment for crystallized IQ did not alter the significant group × RAVLT interaction effects (all *p* < 0.03) or the associations between hippocampal RNI and both immediate and delayed recall, as well as between hippocampal RND and immediate recall in males with mTBI (all *p* < 0.04). The association between hippocampal RND and delayed recall was attenuated to trend level after IQ adjustment (*β* = 0.11, *p* = 0.09), although the effect size remained comparable to the primary analysis. This attenuation may reflect the greater reliance of delayed recall on broader cognitive abilities captured by IQ. However, this result should be interpreted in the context of the reduced sample size due to missing IQ data.

## 4 Discussion

Applying an advanced diffusion MRI technique, this study investigated the microstructure of the hippocampus and associated white matter bundles in young adolescents with and without a lifetime history of mTBI. Male adolescents with a lifetime history of mTBI exhibited greater hippocampal RND than males without a history of mTBI. No such difference was observed in females. Although hippocampal RNI did not survive correction for multiple comparisons in group-level analyses, it showed a similar male-specific pattern. Furthermore, greater hippocampal RNI and RND were associated with better performance on verbal memory tests in male adolescents with a lifetime history of mTBI, suggesting that increased restricted diffusion in the hippocampus might reflect adaptive neuroplastic processes. Our findings could suggest the presence of microstructural reorganization in the hippocampus after mTBI in young adolescents and possible sex differences in the recovery mechanisms following mTBI.

The anatomical substrates underlying the observed increase in restricted diffusion remain to be determined; however, preclinical studies suggest several neural responses following mTBI that may contribute. One potential mechanism is extensive neuronal plasticity after injury, including processes such as dendritic remodeling, synaptogenesis, and axonal sprouting (Hutchinson et al., 2018; Yeh et al., 2022). These reparative processes are known to be regulated by the brain cellular environment that promotes or inhibits neurite growth (Werner and Stevens, 2015) and have been suggested to assist functional recovery after injury (Hutchinson et al., 2018). Preclinical rat models of TBI have demonstrated associations between neuronal remodeling and diffusion metrics, particularly in the hippocampus (Hutchinson et al., 2012, 2018; Kharatishvili et al., 2007; Laitinen et al., 2010; Sierra et al., 2015). The notable findings from these studies include increased anisotropy measured at a 0.75 mm slice thickness scale in association with hippocampal mossy fiber sprouting following brain injury (Laitinen et al., 2010; Sierra et al., 2015). These findings raise the possibility that subtle microstructural alterations at the cellular level may, under certain conditions, be reflected in directional diffusion measures acquired at comparatively coarser *in vivo* resolutions. However, the extent to which these cellular-scale processes contribute to diffusion signals measured at spatial resolutions typical of human imaging (e.g., ~1.7 mm isotropic) remains uncertain; therefore, interpretations linking these findings directly to mossy fiber sprouting should be made with caution. Dendritic remodeling may also increase intracellular space, potentially contributing to increases in restricted isotropic diffusion (White et al., 2013), suggesting that injury-related neurite reorganization may influence both anisotropic and isotropic diffusion components. Notably, the hippocampus comprises heterogeneous tissue elements, including densely packed neuronal cell bodies, elaborate dendritic arbors, and relatively coherent axonal bundles such as the fimbria. Dendritic and axonal reorganization within these distinct compartments may influence both restricted isotropic and directional diffusion components. The relative contribution of these structural elements to the observed RND increase cannot be determined from the present data and will require further investigation.

It has also been reported that the changes in restricted diffusion in the hippocampus may be related to the glial scar formed after injury. Histological analyses indicate that increases in cortical anisotropy following TBI can be associated with the formation of a glial scar (Budde et al., 2011; Laitinen et al., 2015; Sierra et al., 2015; Wiegand et al., 2022). The glial scar within the injured cortex typically forms a highly oriented and elongated structure, it is possible that this type of reparative process may result in increased anisotropy (Budde et al., 2011), a process which may have partly affected our results.

Microstructural alterations were identified only in the hippocampus, while no significant RND changes were observed in other white matter structures. One potential explanation for this regional specificity is the relatively high plasticity of the hippocampus and its distinct microenvironment (Leuner and Gould, 2010). A more plastic or growth-permissive environment may facilitate regenerative responses following injury. Supporting this idea, an experimental study demonstrated that treatments promoting neurotrophin and neuronal growth factor production enhance axonal regeneration and increase anisotropy following brain injury (Jiang et al., 2011). In line with this possibility, rodent resting-state functional MRI studies have reported increased functional connectivity at 4 weeks post-injury in several regions, including the hippocampus (Harris et al., 2016), suggesting the potential for continued reorganization in this region. Methodological factors should also be considered, as subtle abnormalities in larger white matter tracts may be more difficult to detect than those in smaller structures such as the hippocampus, potentially limiting sensitivity to tract-level changes. Additionally, the hippocampus is known to be highly sensitive to secondary pathophysiological cascades following injury, including excitotoxicity, mitochondrial dysfunction, and inflammation (Bigler, 2023). Whether this heightened susceptibility contributes to exaggerated or region-specific microstructural responses following injury remains unclear and warrants further investigation.

The present findings indicate a sex-specific pattern in hippocampal microstructure, with elevated restricted diffusion observed only in male adolescents with a history of mTBI. This male-specific result does not appear to reflect differences in injury-related characteristics, as we found no sex differences in age at injury, time since injury, symptoms, and injury mechanism. Instead, the pattern may point to sex-dependent neurobiological mechanisms that influence how the hippocampus responds to mTBI during early adolescence.

Further investigations are required to clarify the exact mechanisms explaining sex differences in the RSI metrics changes after mTBI, however, there is accumulating evidence indicating sexual dimorphism in response to brain injury. First, an *in vitro* experimental study demonstrated that females have smaller axons and fewer microtubules than males, placing females at risk for more widespread axonal pathology than males subjected to the same injury (Dolle et al., 2018). The more extensive axonal damage found in females has also been suggested to account for the longer duration needed for recovery after TBI (Song et al., 2024). Additionally, studies using a juvenile mouse model of TBI found that male mice display faster and greater neurological recovery, characterized by more rapid return of synaptic function (White et al., 2017), lower inflammatory cytokine expression, and higher microglial/macrophage accumulation (Newell et al., 2020), during the acute to subacute stages after brain injury compared with female mice. Such differences during the early post-injury stages may influence brain responses to mTBI and may partly account for the more pronounced increase in the RSI metrics observed in males. The observed sex differences could also be related to differences in regional response to injury. Previous rodent studies have shown that neuronal and axonal regeneration after TBI in females is evident in the cortex, while changes in the hippocampus are less noticeable when compared to males (Mychasiuk et al., 2016; Rubin and Lipton, 2019), suggesting that sex differences may be region-specific.

It has been suggested that sex hormones, such as progesterone and estrogen, may exert neuroprotective effects and contribute to sex differences in both susceptibility to and recovery from brain injury (Gupte et al., 2019; Rubin and Lipton, 2019). We therefore conducted a sensitivity analysis controlling for current pubertal status (Tanner stage), and found the results remained unchanged, suggesting that it is unlikely that current sex hormone levels influenced the sex-dependent results of this study. However, although most of our participants experienced mTBI before their pubertal development, we acknowledge that this study did not assess hormonal levels at the time of brain injury, and it will be important for future studies to precisely evaluate any hormonal influences on the time course of recovery.

Finally, we found that among male adolescents with a history of mTBI, greater hippocampal restricted diffusion was associated with better immediate and delayed recall scores. Notably, this relationship differed significantly from that observed in adolescents without TBI. These findings suggest that memory performance may be more tightly coupled to hippocampal microstructural properties following injury, potentially reflecting an increased reliance on hippocampal integrity in adolescents with mTBI. Together with elevated restricted diffusion in the mTBI group, this association lends some support for the hypothesis that increased restricted diffusion may reflect a reparative or adaptive neuroplastic process. However, because pre-injury data on cognitive function was not available, it remains unclear whether the observed association reflects neurophysiological recovery following injury or pre-existing cognitive advantages that may have facilitated better recovery outcomes. Future longitudinal studies are needed to clarify this relationship and to determine whether similar mechanisms may underlie the relatively slower recovery of neurocognitive functions, including memory, reported in female children and adolescents with mTBI (Gauvin-Lepage et al., 2019). At the same time, although increased hippocampal RND was positively associated with memory performance in males with mTBI, it remains uncertain whether this pattern reflects true functional recovery or a compensatory process that could signal later vulnerability. Longitudinal follow-up studies will therefore be necessary to determine whether elevated RND predicts favorable recovery trajectories or increased risk for later neurocognitive difficulties.

The elevated hippocampal RND observed in male adolescents with mTBI is not consistent with findings from adult studies showing hippocampal microstructural impairments (e.g., increased mean diffusivity, reduced fractional anisotropy) in chronic mTBI (Leh et al., 2017). This discrepancy may, in part, reflect developmental differences, as recovery in the developing brain occurs alongside dynamic developmental processes, such as myelination, synaptogenesis, and synaptic pruning, which may mask or interact with injury-related effects. Additionally, an adult study in chronic mTBI reported hippocampal hypersynchrony with other cortical regions, which the authors interpreted as a compensatory response to connectivity loss or dysregulation in other areas (Venkatesan et al., 2015). Future multimodal imaging studies could clarify whether such functional changes parallel the hippocampal microstructural alterations and memory outcomes observed in the present study.

In subgroup analyses examining injury timing, age at injury was not associated with memory performance in males. In contrast, among females, younger age at injury (i.e., 0–7 years) was associated with lower memory performance. This sex-specific pattern may be consistent with developmental models emphasizing sensitive periods rather than linear age effects (Zamani et al., 2020). According to this framework, the impact of brain injury depends on whether it occurs during a phase of rapid maturation of the affected function. Early childhood may represent such a sensitive window for verbal memory processes in females, given evidence of earlier or more rapid language-related development in girls during the preschool and early school years (Kaushanskaya et al., 2013). Accordingly, mTBI during this period may confer greater vulnerability in females. This finding does not necessarily imply greater overall risk in females, but rather suggests a potential interaction between injury timing and neurodevelopmental stage. Longitudinal studies will be needed to determine whether these memory differences reflect persistent impairment or delayed maturation followed by eventual catch-up. Additionally, although no corresponding neural correlates were identified in the present study, these findings raise questions about potential sex differences in the maturation of large-scale memory-related networks beyond the hippocampus and the focal white matter tracts examined here, which should be examined in future longitudinal and multimodal imaging studies.

A major strength of the current study is the use of neuroimaging data from a large sample of young adolescents. We restricted participants’ age range to 11–12 years and thus increased the sensitivity of the analyses by minimizing potential developmental-stage variability. However, several limitations should be noted. First, the lifetime history of mTBI was obtained from retrospective parent reports, which may have missed additional injuries (e.g., incidents not reported to an adult) and may be subject to recall bias, potentially leading to misclassification. Second, the age at injury in the mTBI group ranged widely from 0–12 years, although controlling for age at injury did not alter the results (Supplementary Table 7). Third, the present analysis was cross-sectional, and the ABCD Study data used did not include information on acute post-injury symptoms or a reliable estimate of the total number of injuries, highlighting the need for future follow-up studies with more refined designs to better understand the impact of mTBI on neurocognitive development. Fourth, data on pre-injury clinical characteristics were not available, limiting causal interpretation of the correlations between hippocampal restricted diffusion and memory performance. Fifth, the ABCD Study implemented harmonized imaging protocols across scanner platforms to reduce scanner-related variability (Casey et al., 2018), and scanner effects were modeled as random effects in the present analyses to account for potential residual confounding. However, residual variability related to scanner differences cannot be entirely ruled out. Finally, hippocampal subfields, including densely packed axonal structures (e.g., fimbria) and other subfields (e.g., CA2/3, dentate gyrus), may differentially contribute to RND changes after TBI. Future studies that quantify diffusion signals at the subfield level could help distinguish mTBI-related alterations across subfields with distinct histological features.

## 5 Conclusion

This study identified hippocampal microstructural differences between young adolescents with and without a history of mTBI. Given the cross-sectional design of the analyses, these findings should be considered preliminary and require longitudinal follow-up studies to determine whether increased hippocampal RND predicts recovery trajectories or later neurocognitive risk. Sex-specific findings highlight differences in hippocampal features associated with mTBI between males and females. This study, which used an advanced diffusion MRI technique, may provide a foundation for further work aimed at elucidating recovery mechanisms in the developing brain following mTBI.

## Data Availability

Publicly available datasets were analyzed in this study. This data can be found at: NIMH Data Archive (https://doi.org/10.15154/z563-zd24).
