# Supplementary material for: Sex-specific hippocampal microstructural alterations in 11–12-year-old adolescents with a history of mild traumatic brain injury
Source: Front Behav Neurosci. 2026 May 8;20:1766772. doi: 10.3389/fnbeh.2026.1766772 (PMC13194133; doi:10.3389/fnbeh.2026.1766772)
Supplement: Supplementary file 1 [file Data_Sheet_1.pdf]

Supplementary Material

Ma et al., Sex-specific hippocampal microstructural alterations in 11–12-year-old adolescents with a history of mild traumatic brain injury

**Contents**		**Page**
Supplementary Table 1	Differences in subcortical restricted diffusion between adolescents with and without mTBI, by sex.	2
Supplementary Table 2	Differences in restricted normalized directional diffusion in white matter tracts between adolescents with and without mTBI, by sex.	3
Supplementary Table 3	Differences in subcortical volumes between adolescents with and without mTBI, by sex.	4
Supplementary Table 4	Subgroup analysis by LOC status.	5
Supplementary Table 5	Subgroup analysis by age-at-injury.	6
Supplementary Table 6	Subgroup analysis by emergency department (ED) visit history.	7
Supplementary Table 7	Associations between hippocampal diffusion measures and memory performance in adolescents with mTBI, additionally controlling for age-at-injury.	8

Supplementary Table 1. Differences in subcortical restricted diffusion between adolescents with and without mTBI, by sex.

	Male				Female				$P_{Gr×Sex}$
	B (95% CI)	z	P_{uncorr}	P_{corr}	*B* (95% CI)	z	P_{uncorr}	P_{corr}	
RNI									
Hippocampus	0.14 (0.02, 0.26)	2.3	0.02	0.15	0.03 (-0.13, 0.19)	0.4	0.71	1.00	0.18
Amygdala	0.06 (-0.05, 0.17)	1.0	0.31	1.00	0.07 (-0.08, 0.22)	0.9	0.38	1.00	0.88
Thalamus	0.02 (-0.07, 0.11)	0.4	0.68	1.00	-0.07 (-0.20, 0.05)	-1.2	0.24	1.00	0.24
Caudate	0.07 (-0.05, 0.19)	1.2	0.24	1.00	-0.12 (-0.28, 0.03)	-1.5	0.13	0.90	0.07
Putamen	0.04 (-0.08, 0.15)	0.7	0.52	1.00	-0.02 (-0.17, 0.12)	-0.3	0.77	1.00	0.51
Pallidum	0.03 (-0.06, 0.13)	0.7	0.50	1.00	-0.06 (-0.18, 0.06)	-1.0	0.33	1.00	0.23
Nucleus accumbens	-0.004 (-0.11, 0.10)	-0.1	0.95	1.00	-0.11 (-0.25, 0.03)	-1.5	0.13	0.93	0.25
RND									
Hippocampus	0.18 (0.06, 0.30)	3.0	0.003	0.02	0.12 (-0.03, 0.27)	1.6	0.12	0.81	0.55
Amygdala	0.04 (-0.07, 0.15)	0.7	0.47	1.00	-0.03 (-0.18, 0.11)	-0.5	0.62	1.00	0.37
Thalamus	0.04 (-0.08, 0.16)	0.7	0.50	1.00	-0.04 (-0.20, 0.11)	-0.6	0.57	1.00	0.47
Caudate	0.07 (-0.03, 0.17)	1.3	0.20	1.00	-0.04 (-0.16, 0.09)	-0.6	0.57	1.00	0.30
Putamen	0.05 (-0.05, 0.14)	1.0	0.33	1.00	-0.02 (-0.14, 0.10)	-0.3	0.76	1.00	0.49
Pallidum	-0.02 (-0.14, 0.10)	-0.3	0.76	1.00	0.04 (-0.11, 0.19)	0.5	0.64	1.00	0.69
Nucleus accumbens	0.05 (-0.05, 0.15)	0.9	0.36	1.00	-0.06 (-0.19, 0.06)	-1.0	0.32	1.00	0.16

Note: Group differences were tested using a mixed-effects regression model that included age, parental education, race/ethnicity, and total intracranial volume as covariates, and scanner and study site as random effects. Both uncorrected (P_{uncorr}) and Bonferroni-corrected (P_{corr}) *P* values are presented. Diffusion measures were standardized using the sample mean and standard deviation prior to analysis to facilitate comparisons across brain regions and between sexes. Abbreviations: mTBI, mild traumatic brain injury; CI, confidence interval; RNI, restricted normalized isotropic diffusion; RND, restricted normalized directional diffusion.

Supplementary Table 2. Differences in restricted normalized directional diffusion in white matter tracts between adolescents with and without mTBI, by sex.

	Male				Female				$P_{Gr \times Sex}$
	B (95% CI)	*z*	P_{uncorr}	P_{corr}	*B* (95% CI)	*z*	P_{uncorr}	P_{corr}	
Hippocampal and memory-related white matter tracts									
Fornix	0.002 (-0.13, 0.13)	0.04	0.97	1.00	-0.03 (-0.19, 0.12)	-0.4	0.68	1.00	0.88
Hippocampal cingulum	0.06 (-0.04, 0.17)	1.2	0.24	1.00	-0.03 (-0.16, 0.10)	-0.4	0.67	1.00	0.32
Uncinate fasciculus	-0.001 (-0.11, 0.10)	-0.03	0.98	1.00	0.02 (-0.10, 0.14)	0.3	0.76	1.00	0.80
Inferior longitudinal fasciculus	0.07 (-0.05, 0.18)	1.2	0.24	1.00	-0.06 (-0.19, 0.08)	-0.9	0.39	1.00	0.12
Arcuate fasciculus	0.04 (-0.07, 0.15)	0.7	0.50	1.00	0.0003 (-0.13, 0.13)	0.0	1.00	1.00	0.71
Comparison tract									
Corpus callosum	0.05 (-0.05, 0.16)	1.0	0.32	1.00	0.08 (-0.05, 0.20)	1.2	0.24	1.00	0.69

Note: Group differences were tested using a mixed-effects regression model that included age, parental education, race/ethnicity, and total intracranial volume as covariates, and scanner and study site as random effects. Both uncorrected (P_{uncorr}) and Bonferroni-corrected (P_{corr}) *P* values are presented. Diffusion measures were standardized prior to analysis using the sample mean and standard deviation to facilitate comparisons across white matter tracts and between sexes. Abbreviations: mTBI, mild traumatic brain injury; CI, confidence interval.

Supplementary Table 3. Differences in subcortical volumes between adolescents with and without mTBI, by sex.

	Male				Female				$P_{\mathrm{Gr×Sex}}$
	B (95% CI)	z	P_{uncorr}	P_{corr}	*B* (95% CI)	z	P_{uncorr}	P_{corr}	
Hippocampus	0.07 (-0.03, 0.18)	1.3	0.18	1.00	-0.02 (-0.14, 0.10)	-0.3	0.78	1.00	0.35
Amygdala	0.07 (-0.04, 0.18)	1.3	0.21	1.00	0.004 (-0.12, 0.13)	0.1	0.95	1.00	0.52
Thalamus	0.03 (-0.07, 0.12)	0.6	0.56	1.00	0.08 (-0.03, 0.18)	1.4	0.15	1.00	0.47
Caudate	0.01 (-0.11, 0.13)	0.2	0.87	1.00	0.004 (-0.13, 0.14)	0.1	0.95	1.00	0.94
Putamen	0.01 (-0.10, 0.13)	0.2	0.82	1.00	-0.02 (-0.15, 0.11)	-0.3	0.75	1.00	0.70
Pallidum	-0.004 (-0.12, 0.11)	-0.1	0.95	1.00	-0.05 (-0.17, 0.08)	-0.7	0.47	1.00	0.58
Nucleus accumbens	0.04 (-0.07, 0.15)	0.7	0.47	1.00	-0.09 (-0.23, 0.04)	-1.3	0.18	1.00	0.14

Note: Group differences were tested using a mixed-effects regression model that included age, parental education, race/ethnicity, and total intracranial volume as covariates, and scanner and study site as random effects. Both uncorrected (P_{uncorr}) and Bonferroni-corrected (P_{corr}) *P* values are presented. Volume measures were standardized using the sample mean and standard deviation prior to analysis to facilitate comparisons across brain regions and between sexes. Abbreviations: mTBI, mild traumatic brain injury; CI, confidence interval.

Supplementary Table 4. Subgroup analysis by LOC status.

	Male						Female					
	No TBI vs. mTBI without LOC			No TBI vs. mTBI with LOC			No TBI vs. mTBI without LOC			No TBI vs. mTBI with LOC		
	B (95% CI)	z	*P*	*B* (95% CI)	z	*P*	*B* (95% CI)	z	*P*	*B* (95% CI)	z	*P*
Hippocampal RNI	0.13 (-0.04, 0.30)	1.5	0.15	0.16 (-0.01, 0.32)	1.9	0.06	-0.05 (-0.29, 0.19)	-0.4	0.69	0.08 (-0.12, 0.28)	0.8	0.43
Hippocampal RND	0.16 (-0.003, 0.33)	1.9	0.054	0.20 (0.04, 0.37)	2.5	0.01	0.04 (-0.20, 0.27)	0.3	0.76	0.17 (-0.01, 0.36)	1.8	0.07
RAVLT outcomes												
Verbal learning	-0.46 (-2.18, 1.25)	-0.5	0.60	1.03 (-0.65, 2.71)	1.2	0.23	0.14 (-2.21, 2.49)	0.1	0.91	0.49 (-1.42, 2.41)	0.5	0.61
New learning	-0.19 (-0.45, 0.08)	-1.4	0.17	0.21 (-0.05, 0.47)	1.6	0.12	-0.04 (-0.42, 0.33)	-0.2	0.82	0.02 (-0.29, 0.32)	0.1	0.91
Immediate recall	-0.31 (-0.83, 0.21)	-1.2	0.24	0.11 (-0.40, 0.62)	0.4	0.67	-0.11 (-0.83, 0.60)	-0.3	0.76	0.22 (-0.37, 0.80)	0.7	0.47
Delayed recall	-0.15 (-0.71, 0.41)	-0.5	0.59	-0.04 (-0.60, 0.51)	-0.2	0.88	-0.45 (-1.22, 0.33)	-1.1	0.26	0.38 (-0.24, 1.01)	1.2	0.23
Consolidated information, %	-0.01 (-0.05, 0.03)	-0.7	0.48	-0.04 (-0.08, 0.001)	-1.9	0.06	-0.03 (-0.09, 0.02)	-1.1	0.26	0.004 (-0.04, 0.05)	0.2	0.86

Note: Group differences were tested using a mixed-effects regression model that included age, parental education, race/ethnicity, and total intracranial volume as covariates. For diffusion measures, scanner and study site were included as random effects; for RAVLT outcomes, study site was included as a random effect. Abbreviations: LOC, loss of consciousness; mTBI, mild traumatic brain injury; CI, confidence interval; RNI, restricted normalized isotropic diffusion; RND, restricted normalized directional diffusion; RAVLT, Rey Auditory Verbal Learning Test.

Supplementary Table 5. Subgroup analysis by age-at-injury.

	Male						Female					
	No TBI vs. mTBI (0–7 years)			No TBI vs. mTBI (8–12 years)			No TBI vs. mTBI (0–7 years)			No TBI vs. mTBI (8–12 years)		
	B (95% CI)	z	*P*	*B* (95% CI)	z	*P*	*B* (95% CI)	z	*P*	*B* (95% CI)	z	*P*
Hippocampal RNI	0.02 (-0.20, 0.25)	0.2	0.83	0.26 (0.01, 0.51)	2.0	0.04	-0.12 (-0.42, 0.19)	-0.7	0.46	0.06 (-0.33, 0.46)	0.3	0.75
Hippocampal RND	0.15 (-0.07, 0.37)	1.4	0.18	0.18 (-0.07, 0.43)	1.4	0.16	-0.08 (-0.37, 0.21)	-0.5	0.59	0.23 (-0.15, 0.60)	1.2	0.23
RAVLT outcomes												
Verbal learning	-0.88 (-3.19, 1.42)	-0.8	0.45	0.05 (-2.44, 2.54)	0.04	0.97	-0.73 (-3.71, 2.25)	-0.5	0.63	1.47 (-2.30, 5.24)	0.8	0.44
New learning	-0.31 (-0.67, 0.04)	-1.7	0.09	-0.04 (-0.42, 0.35)	-0.2	0.86	-0.07 (-0.54, 0.40)	-0.3	0.78	0.01 (-0.59, 0.60)	0.03	0.98
Immediate recall	-0.41 (-1.10, 0.28)	-1.2	0.24	-0.18 (-0.94, 0.57)	-0.5	0.64	-0.62 (-1.52, 0.28)	-1.4	0.18	0.71 (-0.43, 1.84)	1.2	0.22
Delayed recall	-0.19 (-0.93, 0.56)	-0.5	0.63	-0.08 (-0.89, 0.73)	-0.2	0.85	-1.06 (-2.02, -0.10)	-2.2	0.03	0.62 (-0.66, 1.90)	1.0	0.34
Consolidated information, %	-0.01 (-0.07, 0.04)	-0.5	0.60	-0.01 (-0.07, 0.04)	-0.4	0.68	-0.08 (-0.15, -0.01)	-2.2	0.03	0.04 (-0.05, 0.14)	0.9	0.35

Note: Group differences were tested using a mixed-effects regression model that included age, parental education, race/ethnicity, and total intracranial volume as covariates. For diffusion measures, scanner and study site were included as random effects; for RAVLT outcomes, study site was included as a random effect. Abbreviations: mTBI, mild traumatic brain injury; CI, confidence interval; RNI, restricted normalized isotropic diffusion; RND, restricted normalized directional diffusion; RAVLT, Rey Auditory Verbal Learning Test.

Supplementary Table 6. Subgroup analysis by emergency department (ED) visit history.

	Male						Female					
	No TBI vs. mTBI (ED-)			No TBI vs. mTBI (ED+)			No TBI vs. mTBI (ED-)			No TBI vs. mTBI (ED+)		
	B (95% CI)	z	*P*	*B* (95% CI)	z	*P*	*B* (95% CI)	z	*P*	*B* (95% CI)	z	*P*
Hippocampal RNI	0.13 (-0.05, 0.30)	1.5	0.15	0.15 (-0.01, 0.31)	1.9	0.06	-0.02 (-0.26, 0.22)	-0.2	0.84	0.07 (-0.13, 0.27)	0.7	0.52
Hippocampal RND	0.12 (-0.05, 0.29)	1.4	0.17	0.24 (0.08, 0.40)	3.0	0.003	-0.02 (-0.25, 0.21)	-0.2	0.85	0.22 (0.03, 0.41)	2.2	0.03
RAVLT outcomes												
Verbal learning	0.48 (-1.25, 2.21)	0.6	0.59	0.13 (-1.53, 1.80)	0.2	0.87	0.89 (-1.43, 3.22)	0.8	0.45	-0.02 (-1.95, 1.92)	-0.02	0.99
New learning	0.07 (-0.20, 0.34)	0.5	0.61	-0.04 (-0.29, 0.22)	-0.3	0.78	-0.11 (-0.48, 0.26)	-0.6	0.56	0.06 (-0.24, 0.37)	0.4	0.68
Immediate recall	-0.18 (-0.71, 0.35)	-0.7	0.51	-0.02 (-0.53, 0.48)	-0.1	0.93	0.57 (-0.13, 1.28)	1.6	0.11	-0.25 (-0.84, 0.34)	-0.8	0.40
Delayed recall	-0.21 (-0.78, 0.36)	-0.7	0.47	0.004 (-0.54, 0.55)	0.02	0.99	0.23 (-0.53, 1.00)	0.6	0.56	-0.06 (-0.69, 0.58)	-0.2	0.86
Consolidated information, %	-0.03 (-0.07, 0.01)	-1.5	0.14	-0.02 (-0.06, 0.02)	-1.2	0.25	-0.005 (-0.06, 0.05)	-0.2	0.87	-0.01 (-0.06, 0.03)	-0.6	0.55

Note: Group differences were tested using a mixed-effects regression model that included age, parental education, race/ethnicity, and total intracranial volume as covariates. For diffusion measures, scanner and study site were included as random effects; for RAVLT outcomes, study site was included as a random effect. Abbreviations: mTBI, mild traumatic brain injury; CI, confidence interval; RNI, restricted normalized isotropic diffusion; RND, restricted normalized directional diffusion; RAVLT, Rey Auditory Verbal Learning Test.

Supplementary Table 7. Associations between hippocampal diffusion measures and memory performance in adolescents with mTBI, additionally controlling for age-at-injury.

RAVLT outcomes	Male			Female		
	β (95% CI)	z	*P*	*β* (95% CI)	z	*P*
Hippocampal RNI						
Verbal learning	0.12 (-0.01, 0.24)	1.9	0.06	0.03 (-0.13, 0.19)	0.4	0.72
New learning	0.04 (-0.09, 0.17)	0.6	0.56	0.03 (-0.13, 0.20)	0.4	0.70
Immediate recall	0.20 (0.08, 0.33)	3.2	0.001	0.10 (-0.06, 0.25)	1.2	0.23
Delayed recall	0.21 (0.09, 0.33)	3.4	<0.001	0.05 (-0.11, 0.22)	0.6	0.52
Consolidated information, %	0.14 (0.01, 0.26)	2.2	0.03	0.06 (-0.11, 0.22)	0.7	0.49
Hippocampal RND						
Verbal learning	0.08 (-0.04, 0.19)	1.3	0.19	-0.04 (-0.19, 0.10)	-0.6	0.54
New learning	0.003 (-0.11, 0.12)	0.1	0.96	0.03 (-0.12, 0.17)	0.3	0.74
Immediate recall	0.15 (0.04, 0.26)	2.6	0.01	-0.07 (-0.21, 0.07)	-1.0	0.30
Delayed recall	0.12 (0.01, 0.23)	2.1	0.04	-0.05 (-0.19, 0.10)	-0.6	0.52
Consolidated information, %	0.09 (-0.02, 0.20)	1.6	0.12	0.02 (-0.13, 0.16)	0.2	0.81

Note: Correlations were tested using a mixed-effects regression model, including age, parental education, race/ethnicity, age at injury, and total intracranial volume as covariates, and scanner and study site as random effects.
Abbreviations: mTBI, mild traumatic brain injury; RAVLT, Rey Auditory Verbal Learning Test; CI, confidence interval; RNI, restricted normalized isotropic diffusion; RND, restricted normalized directional diffusion.
